# A reform of value-added taxes on foods can have health, environmental and economic benefits in Europe

**DOI:** 10.1038/s43016-024-01097-5

**Published:** 2025-01-09

**Authors:** Marco Springmann, Eugenia Dinivitzer, Florian Freund, Jørgen Dejgård Jensen, Clara G. Bouyssou

**Affiliations:** 1 Environmental Change Institute, University of Oxford, Oxford, UK; 2 Institute for Global Health, University College London, London, UK; 3 Johann Heinrich von Thünen Institute – Federal Research Institute for Rural Areas, Forestry and Fisheries, Institute of Market Analysis, Braunschweig, Germany; 4 Department of Food and Resource Economics, University of Copenhagen, Copenhangen, Denmark

**Keywords:** Economics, Economics

## Abstract

Fiscal policies can provide important incentives for encouraging the dietary changes needed to achieve global policy targets. Across Europe, the foods relevant to health and the environment often incur reduced but non-zero value-added tax (VAT) rates at about half the maximum rates, which allows for providing both incentives and disincentives. Integrating economic, health and environmental modelling, we show that reforming VAT rates on foods, including increasing rates on meat and dairy, and reducing VAT rates on fruits and vegetables can improve diets and result in health, environmental and economic benefits in most European countries. The health improvements were primarily driven by reductions in VAT rates on fruits and vegetables, whereas most of the environmental and revenue benefits were driven by increased rates on meat and dairy. Our findings suggest that differentiating VAT rates based on health and environmental considerations can support changes towards healthier and more sustainable diets in Europe.

## Abstract (web summary)

Fiscal incentives on consumption can encourage dietary changes towards healthier and more sustainable foods. Integrated modelling reveals the potential health, environmental and economic impacts of aligning VAT rates on food with health and environmental considerations in Europe.

## Main

Diets in most high-income countries are both unhealthy and unsustainable^1^. Dietary risks, including low intake of fruits, vegetables, legumes, nuts and whole grains, and high intake of red meat, processed meat and sugar-sweetened beverages, are a leading cause for premature mortality. They are responsible for up to one in four deaths globally^2^, and for costs projected to reach US$1.3 trillion in 2030^3,4^. Current diets are also a major driver of climate change, responsible for about a third of global greenhouse gas (GHG) emissions^5^, and costs projected to reach US$1.7 trillion in 2030^3,4^, with the majority of climate change impacts attributed to animal-source foods^6–8^. Without dedicated changes towards healthier and more plant-based diets, there is little chance of staying below a global warming of 2 °C and avoiding dangerous levels of climate change^6^, or for reducing the many hidden costs of the food system^9^.

Providing fiscal incentives to consumers that would encourage dietary changes towards healthier and more sustainable diets is seen as an important and effective policy measure to transform diets and food systems^10–12^. Increasing prices of unhealthy and unsustainable foods and decreasing them on healthier and more sustainable foods is in line with economic reasoning and addresses market failures from costs related to food consumption (for example, damage costs from climate change, and care and labour costs from diet-related ill health) that are born by society but not reflected in the market prices of foods, something that results in overconsumption of unhealthy and unsustainable foods^13^.

Although fiscal incentives are less restrictive than mandates and bans, they can be politically controversial when added as new measures^14–17^. An alternative to introducing new tax and subsidy instruments is to reform the current system of taxation. Value-added tax (VAT) is one of the major existing tax systems, accounting for over a fifth of public revenues in the European Union (EU)^18^. VAT is levied on the price of a product or service at each stage of production, distribution and sale to the final consumer. Because it is based on the location of the consumer and applied to sales prices, it functions as a tax on consumption, which makes it relevant for addressing consumption-related impacts.

At the political level, there has been increased debate on pursuing VAT reform for addressing social and environmental objectives related to food. As an outcome of this, the European Parliament recently adopted a new directive that allows EU member states to differentiate VAT rates to pursue social and environmental objectives^18^. The intention of this measure is to bring VAT regulation in line with the European Union’s Green Deal and its Farm to Fork strategy, which includes the target of creating a healthy food environment by supporting healthy and sustainable food choices^19^. However, the directive also highlighted that there is a lack of evidence on assessing the effectiveness of such a measure.

Here we estimate the potential health, environmental and economic impacts of aligning VAT rates on food with health and environmental considerations in Europe. We analysed three main policy scenarios: (1) zero VAT rates on fruits and vegetables (F&V), (2) increasing VAT rates on meat and dairy (M&D) to the maximum rates in each country, and (3) a combination of the two. Our regional coverage included all EU countries plus the United Kingdom.

The choice of targeted food groups is in line with health and environmental considerations and the political discussion^13,20^. F&V, as well as legumes and nuts which we include in the same category, have been consistently associated with reductions in diet-related disease risk^21–23^, and have among the lowest GHG emissions per weight^7,24,25^. In contrast, M&D have the greatest GHG emissions per weight^7,24,25^, are responsible for the majority of food-related GHG emissions within Europe^2,6,7^, and reductions in their intake have either been associated with lower disease risk (red and processed meat) or no health risk (white meat and dairy)^21–23,26^. We considered variations of these policy scenarios in our sensitivity analysis, including limiting increased VAT rates to meat and to red meat.

Our analysis combines health, environmental and economic assessments (Methods). To analyse the demand reactions to changes in VAT rates, we used recently collected data on demand elasticities and adapted those to the European context in an economically consistent manner by using machine-learning and maximum-entropy approaches^27,28^. The demand system resolved three impacts, including how a change in the price of a food commodity affects the demand for that same commodity; how the change in price affects the demand for other commodities that can act as substitutes or complements; and how a change in purchasing power affects overall demand.

For the health assessment, we used a comparative risk assessment that relates changes in diet and weight-related risk factors (for example, increases in F&V intake and reductions in red meat intake) to changes in disease risk and mortality (for example, from coronary heart disease (CHD), stroke and cancer) per year^29–31^. For the environmental assessment, we coupled the changes in demand to a set of region- and commodity-specific environmental footprints for GHG emissions, land use, freshwater use and eutrophication potential associated with nutrient pollution, for example, from the overapplication of fertilizers^7,32^. And for the economic assessment, we coupled the changes in demand to a set of country- and commodity-specific market prices of foods^33,34^.

## Results

**Fig. 1:**
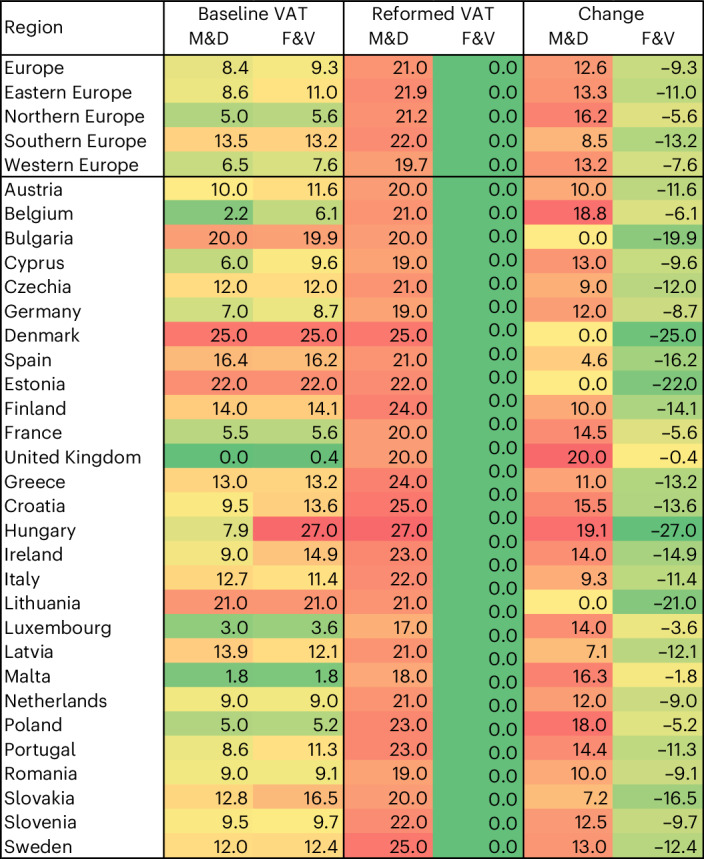
Overview of VAT rates (%) on food categories. The food categories include M&D (composed of beef, lamb, pork, poultry and milk) and F&V (composed of fruits, vegetables, legumes and nuts). The VAT rates include current VAT rates (Baseline VAT), VAT rates in the scenario of VAT reform (Reformed VAT) which consists of combining increased VAT rates on M&D with reduced VAT rates on F&V, and the percentage-point changes in this scenario compared with baseline rates (Change). The colour shading indicates high values in red and low values in green.

VAT rates in Europe differ by region and food group (Fig. 1). Averaged by population, the rates were 8% for M&D, including 8% on beef, lamb, pork and poultry, and 9% on milk and dairy; and were 9% for F&V, including 10% on fruits, and 9% on vegetables, legumes and nuts (Supplementary Table 5). The rates ranged from 0% on M&D and F&V in the United Kingdom to 25% on M&D in Denmark and 27% on F&V in Hungary (Supplementary Fig. 2). More than half of countries (57%, *n* = 16) had similar VAT rates on M&D as on F&V (with a difference in rates <5%), about a third (36%, *n* = 10) had significantly greater VAT rates on F&V, and less than a tenth (7%, *n* = 2; Italy, Latvia) had significantly greater VAT rates on M&D.

Increasing VAT rates on M&D and decreasing VAT rates on F&V impacted the relative prices of foods (Figs. 1 and 3a,b). Prices of M&D increased by 13 percentage points (pp) on average to 21% when taxed at the maximum rate on foods in each country, whereas prices of F&V decreased by 9 pp on average when zero-rated. The largest increases in prices of M&D were for the United Kingdom (20 pp), Hungary (19 pp), Belgium (19 pp) and Poland (18 pp), whereas Lithuania, Estonia, Denmark and Bulgaria already had full VAT rates on all foods and therefore no change in prices. The largest reductions in prices of F&V were for these countries (20–25 pp) and for Hungary (27 pp), whereas the United Kingdom (0 pp), Malta (2 pp), Luxembourg (4 pp) and Poland (5 pp) had the lowest reductions due to low baseline rates.

### Changes in food demand

**Fig. 2:**
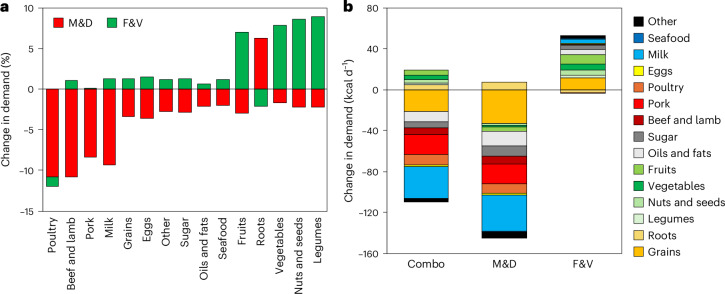
Changes in food demand in the scenarios of VAT reform. The scenarios include increases in VAT rates on M&D to maximum rates in each country, eliminating VAT rates on F&V, and a combination of the two (Combo). **a**, Average percentage changes in food demand by food group across Europe in the Combo scenario, with the contribution from the M&D and the F&V scenarios highlighted. For example, eliminating VAT on F&V increased their demand, but also the demand for foods consumed together with F&V (green bars), whereas increasing VAT on M&D reduced demand for animal products, increased the demand for roots that act as substitute, and reduced the demand for other foods to balance the reduction in spending power. The effect of combining both changes in VAT is represented by the sum of the red and green bars. **b**, Average changes in food demand in terms of kilocalories per person per day (kcal d^−1^) by food group and policy scenario. The coloured panels inside the bars indicate the contribution of each food group to the change in calorie intake, with the sum of all positive and negative contributions indicating the total change in calorie intake.

The VAT-related price changes resulted in changes in food demand (Fig. 2a,b). Levying full VAT rates on M&D decreased the demand for M&D in Europe by 9% (70 grammes per person per day, g d^−1^) on average, most of which stemmed from milk (−49 g d^−1^; 69% of the change), followed by pork (−9 g d^−1^; 13%), poultry (−7 g d^−1^; 10%), and beef and lamb (5 g d^−1^; 8%). The demand for other foods also changed due to substitution and income effects: roots are considered as a replacement and their demand increased (+11 g d^−1^; +6%), whereas the demand for F&V decreased (−13 g d^−1^; −2%) to balance out reductions in spending power. Overall calorie demand decreased by 140 kcal d^−1^ (4%).

Zero-rating VAT on F&V increased the demand for F&V in Europe by 8% (42 g d^−1^) on average, most of which stemmed from vegetables (22 g d^−1^; corresponding to 52% of the total increase in F&V), followed by fruits (18 g d^−1^; 43%), nuts (2 g d^−1^; 4%) and legumes (1 g d^−1^; 2%). The demand for roots decreased slightly (−4 g d^−1^; −2%) due to substitution and income effects, and the demand for most other foods increased slightly (1%) due to income and complementarity effects. Overall calorie demand increased by 50 kcal d^−1^ (1%). Combining the changes in VAT rates on M&D and F&V resulted in similar changes, but with smaller reductions in the demand for M&D (−8.6% versus −9.4%) and lower increases in the demand for F&V (+5.1% versus +7.5%), and with overall calorie demand decreasing by 90 kcal d^−1^ (−3%).

**Fig. 3:**
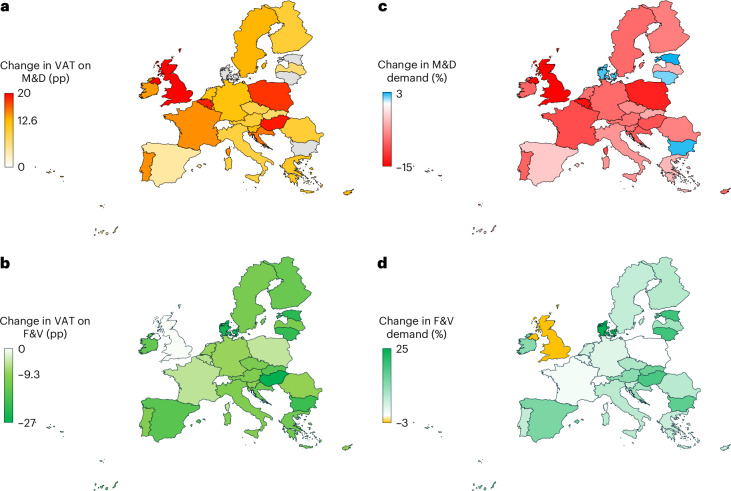
Changes in VAT rates and the related changes in food demand across European countries. **a**–**d**, Changes for the food categories of M&D and F&V in pp for VAT rates (**a**,**b**) and in percent (%) for food demand (**c**,**d**). The changes in demand (**c**,**d**) are for the combined scenario in which VAT rates on M&D are increased and VAT rates on F&V are eliminated.

The combined changes in demand across countries were in line with the regional changes in prices (Fig. 3c–d and Supplementary Fig. 3). The reductions in the demand for M&D were greatest in the United Kingdom (−15%, 111 g d^−1^), Belgium (−14%, 77 g d^−1^) and Poland (−13%, 93 g d^−1^), whereas the increases in demand for F&V were greatest in Denmark (25%, 113 g d^−1^) and Estonia (+19%, 85 g d^−1^). When changes in VAT rates on M&D and F&V were combined, some countries exhibited small increases (1–3%) in M&D (*n* = 4: Bulgaria, Denmark, Estonia, Lithuania) or small decreases (1–3%) in F&D (*n* = 2: United Kingdom, Malta). These impacts occurred where there was no direct price change, but purchasing power effects and complementarity effects with the other food category influenced their demand to result in small changes in the same direction.

### Environmental and health impacts

**Fig. 4:**
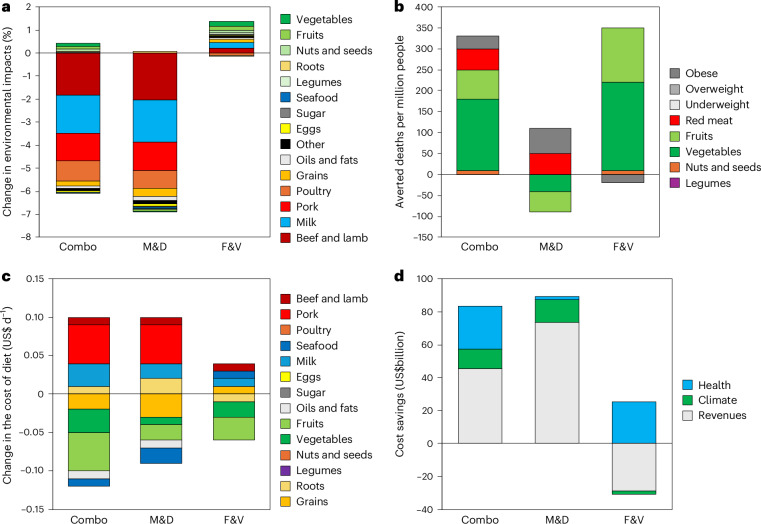
Overview of environmental, health and cost impacts of VAT reform on foods. **a**–**d**, Average changes in environmental impacts (**a**), the number of averted deaths per million people (**b**), changes in the cost of diets (**c**), and cost savings (**d**), each by policy scenario and either food group (**a**–**c**) and cost component (**d**).

The changes in food demand had implications for environmental resource use and pollution. The combined changes in VAT rates resulted in reductions in food-related environmental impacts of 5–6% on average in Europe, including 63 MtCO_2_e in GHG emissions (−6%), 71,000 km^2^ in land use (−6%), 8,240 km^3^ in freshwater use (−5%), and 208 PO_3_^4^e in eutrophication potential (−6%) (Fig. 4a and Supplementary Table 6). Most of these changes were associated with reductions in M&D, including beef and lamb (32% of the total reduction in M&D across the different environmental domains on average), milk (29%), pork (22%) and poultry (15%).

**Fig. 5:**
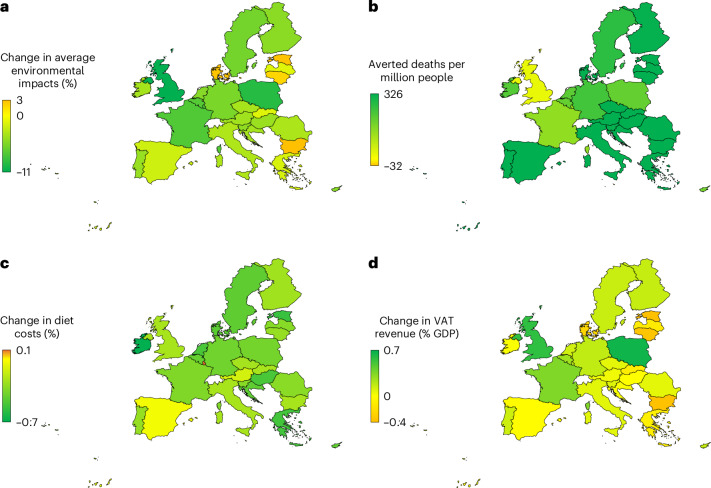
Environmental, health and cost impacts of VAT reform for foods across European countries. **a**–**d**, Changes in averaged environmental impacts (**a**), the number of averted deaths per million people (**b**), the cost of diets (**c**) and VAT revenues (**d**).

Across countries (Fig. 5a), the changes in environmental impacts ranged from reductions of about a tenth in the United Kingdom (−12%) and Poland (−9%) to small increases (2–3%) in Bulgaria, Estonia, Denmark and Lithuania, which were in line with the regional changes in demand for M&D. Limiting VAT reform to increasing rates on M&D resulted in about 1% greater reductions in environmental resource use and pollution on average (7% versus 6%), whilst limiting it to reducing rates on F&V led to net increases in environmental impacts of about 1% on average.

The VAT-related changes in food demand also had impacts on human health by influencing dietary and weight-related risk factors (Fig. 4b and Supplementary Table 7). The combined changes in VAT rates were associated with 170,000 fewer deaths from diet-related diseases in Europe per year (−3%), equivalent to a reduction in mortality of 330 deaths per million people. More than half of these changes (54%) stemmed from fewer coronary heart disease (CHD) deaths, followed by cancer (28%), stroke (15%), type 2 diabetes (2%) and respiratory disease (<1%). By risk factor, three-quarters (74%) of the reductions in mortality were from increased intake of vegetables (51%) and fruits (23%), followed by reduced intake of red meat (15%), and reductions in obesity (10%) that were associated with changes in energy intake.

Across countries (Fig. 5b), the reductions in mortality ranged from 1,390 and 930 per million in Croatia and Hungary to 50 and 30 per million in Malta and the United Kingdom, in line with the regional changes in the demand for F&V. Limiting VAT reform to reducing rates on F&V was associated with similar changes in mortality because greater benefits from increased F&V intake compensated for the lack of change in red meat intake. In contrast, only increasing rates on M&D was associated with neutral impacts because benefits from reductions in red meat and obesity were compensated by reductions in F&V intake due to the reductions on purchasing power.

### Cost impacts

In principle, changes in food prices and demand can impact the cost of diets for consumers, the tax revenues collected by governments, and through their impacts on the environment and health also social costs that are borne by society, for example, as climate-change damages and healthcare costs. In our analysis, the combined changes in VAT rates were not associated with substantial changes in the costs of diets to consumers (−US$0.03 per person per day, US$ d^−1^, on average; −0.3%) because the increased costs of animal products (+US$0.1 d^−1^) were offset by the reduced costs of plant-based foods (−US$0.1 d^−1^) (Fig. 4c).

Despite greater variation at the regional level, the changes in the costs of diets also stayed below 0.5% in each country (Fig. 5c). Limiting VAT reform to increasing rates on animal products or to reducing rates on plant-based foods did not increase the average costs of diets either (−0.2%, −0.1%). This was because lower prices for plant-based foods increased their intake less than their change in price (in line with elasticities of <1) in the latter, and higher prices for animal products shifted demand away from these higher-priced foods and also reduced intake of other foods due to income effects.

In our analysis of tax revenues, comprehensive VAT reform increased food-related tax revenues in Europe by a third (34%) on average, or US$45 billion when adjusted for purchasing power parity (Fig. 4d). The change in revenues contrasted with the change in the cost of diets due to a different tax base that resulted from the induced changes in demand. The change in revenues consisted of a large increase in revenue from animal products (+US$76 billion) that occurred despite the induced demand change, overcompensating the loss of revenue from plant-based foods (−US$30 billion). This was because the demand change away from animal products was less than the increase in VAT rates (in line with elasticities of <1).

By country (Fig. 5d), the changes in revenues ranged from large increases in Poland (+0.7% of national GDP) and the United Kingdom (+0.6% of national GDP) that had small or no baseline taxes on animal products to losses in revenue of 0.3–0.4% of national GDP in Estonia, Denmark, Bulgaria and Lithuania that already taxed animal products at maximum rates. Limiting VAT reform to raising rates on animal products led to larger increases in tax revenues (+56%; US$74 billion), whilst only reducing rates on plant-based foods led to a net loss of a quarter (−22%; US$29 billion) of tax revenues from foods.

There were additional changes in the external costs borne by society that are associated with the food system. Valuing the changes in GHG emissions with estimates of the cost of climate damages resulted in US$12 billion in reduced climate-change costs, and valuing the change in mortality by cause-specific cost-of-illness estimates resulted in US$26 billion in reduced healthcare costs (Fig. 4d). Including the climate and health benefits in the economic valuation almost doubled the net benefits from US$45 billion to US$83 billion (+83%).

The economic impacts also changed at the country level when, in addition to the changes in revenues, the reductions in the costs of climate damages and the reductions in the costs of illness were also accounted for. At the country level, the economic benefits increased to 0.7–0.8% of national GDP in Poland and the United Kingdom, whereas losses were reduced to 0.1–0.2% of national GDP in Bulgaria and Estonia, and below that in Denmark and Lithuania (Supplementary Fig. 4). Limiting VAT reform to raising rates on animal products increased the savings in climate-change damages (to US$14 billion) but substantially reduced the savings in healthcare costs (to US$2 billion), whereas limiting VAT reform to reducing rates on plant-based foods had similar savings in healthcare costs as the combined scenario (US$25 billion), but led to a net increase in the costs of climate change (US$2 million).

## Discussion

Without dietary changes towards healthier and more sustainable diets, there is little chance of achieving the Sustainable Development Goals, staying within global planetary boundaries and avoiding dangerous levels of climate change^1,6^. Fiscal policies can provide important incentives that encourage dietary changes^10–12^. Here we show that reforming VAT rates on foods, including increasing rates on foods with high environmental and health impacts such as M&D, and reducing VAT rates on healthy foods with low environmental impacts such as F&V can help to improve diets and result in health, environmental and cost benefits in most European countries.

Our analysis showed that, in Europe, most foods relevant to health and environmental impacts currently incur reduced but non-zero VAT rates at about half the maximum rate in each country, which allows for providing both incentives and disincentives. Eliminating VAT rates on F&V amounted to a tax reduction of 9 pp on average, ranging from zero in the United Kingdom to 27 pp in Hungary, whereas increasing VAT on M&D represented a tax increase of 13 pp on average, ranging from zero in Bulgaria, Denmark, Estonia and Lithuania to 20 pp in the United Kingdom.

We found that the health improvements were primarily driven by reductions in VAT rates on F&V, whereas most of the environmental and revenue benefits were driven by increased rates on M&D. On average, they amounted to health benefits of 330 averted deaths per million people, reductions in GHG emissions and environmental resource use of 6%, increases in tax revenues of 0.22% of GDP, and reductions in costs to society from ill health and climate damage of 0.18% of GDP. The health benefits were greatest in countries with currently high VAT rates on F&V (for example, Hungary), and the environmental and revenue benefits were greatest in countries with currently low rates on M&D (for example, the United Kingdom).

Combining reduced VAT rates on healthy and sustainable foods with increased rates on unhealthy and unsustainable ones resulted in the best balance of benefits in countries where this was possible. In contrast, limiting VAT reform to reductions on F&V resulted in similar health benefits, but higher environmental impacts and lower tax revenues, whereas solely increasing VAT rates on M&D resulted in higher tax revenues, similar environmental benefits but few health benefits. Combining reductions in VAT rates on F&V with increases in VAT rates only for meat or only for red meat resulted in similar health benefits, but 40–55% less environmental benefits and revenues (Supplementary Table 8).

Our results can be compared to various strands of the literature. Several authors have mentioned using VAT reform to address health or environmental objectives^13,35^, but there have been few quantitative analyses of such proposals. A study by Klenert et al.^36^ has focused on the distributional impacts of VAT reform in Europe, but did not assess the health and environmental impacts and assumed inelastic demand, whereas a study by Oebel et al.^35^ focused on using VAT reform to transform the German food system, but did not conduct a health assessment and did not use a complete and economically consistent demand system. However, the direction of effects of a combination of lowering VAT on (organic) vegetarian foods and raising VAT on conventional meat were similar for environmental impacts and revenues.

Other studies have analysed different fiscal policies such as carbon taxes on foods. For example, Springmann et al.^37^ estimated that levying a carbon price of US$52 tCO_2_e^−1^ on food demand in high-income countries would increase prices of M&D by 8–27% on average, which resulted in emissions reductions of 4% and in about 58,000 averted deaths when revenues were used to subsidize F&V intake. Using the emissions footprint and demand system of our study (Supplementary Table 9), we obtained a similar carbon price (US$63 tCO_2_e^−1^) when approximating the emissions reductions of VAT reform (−6%), but also found that using a social cost of carbon of US$185 tCO_2_e^−1^, which is more in line with recent recommendations^38^, resulted in emissions reductions of 16%. This suggests that VAT reform can account for some of the external costs of climate change, but ideally would be combined with additional measures and policies^29,39,40^.

Politically, the benefits of addressing health and environmental objectives by changes in existing tax systems such as VAT are that they might be less contentious than introducing new and potentially more targeted instruments such as carbon prices^14–16^. Legally, differentiating VAT rates in such a way has recently been made easier within the European Union by a new directive^18^. However, it is within the jurisdiction of each EU country to pass any reform to their specific VAT system. For example, specific discussions of reforming VAT rates on foods are underway in the Netherlands and Germany^20,35^. Our findings suggest that most European countries could benefit in terms of public health, environmental sustainability, and costs and revenues from reforming their VAT rates on foods.

From an economic perspective, each market failure would ideally be addressed by a dedicated policy instrument to minimize trade-offs^41,42^. However, market failures related to food systems are rarely completely independent, given imbalanced diets are associated with a combination of detrimental impacts such as climate change, biodiversity loss and ill health^1,2^. Thus, synergies exist for any one policy that affects dietary demand even though complementary policies might be needed to fully achieve the intended policy objectives. This is especially the case for distributional concerns because increased taxation of foods can have regressive effects when it lacks compensating mechanisms and dietary substitution is difficult^13,36,43–46^. For VAT reform, balancing an increase in VAT for M&D with reductions in VAT for F&V addresses this concern, but additional measures such as rebating a proportion of tax revenues might be needed^36^.

Although our study has several strengths when compared to the literature, such as the use of an economically consistent demand system and its integration with health and environmental assessments, it is also subject to several caveats. First, although our demand system relied on a comprehensive meta-analysis of food demand elasticities, it included a greater number of observations on animal products compared to plant-based foods. We consider the number of observations sufficient for food-group analyses (Supplementary Section 1), but note that integration of more observations for plant-based foods would allow for a more detailed substitution analysis. Second, in our health assessment (Supplementary Section 2), we assumed that the risk–disease relationships describe causal associations, an assumption supported by the existence of statistically significant dose–response relationships in meta-analyses, the existence of plausible biological pathways and supporting evidence from experiments, for example, on intermediate risk factors^21–23,47–50^. However, residual confounding with unaccounted risk factors cannot be ruled out in epidemiological studies.

Third, in our environmental assessment (Supplementary Section 3), we used a regionalized set of environmental footprints derived from a comprehensive meta-analysis of lifecycle assessments. The use of country-specific values would further improve the precision of our assessment, but the available meta-analyses suggest that footprints differ more across food categories than across regions^7,25^. Fourth, we did not explicitly resolve the supply-side responses to changes in demand that can act to dampen the effect on market prices^51^. Economic models (partial and general equilibrium models) exist to study such effects, but their resolution of regions and food commodities is often low, and many employ relatively inelastic demand systems based on outdated elasticities^28,52^. Fifth, we did not consider how VAT reform would interact with other tax policies such as taxes on sugary drinks and carbon taxes in agriculture. Analysing the interactions between potentially overlapping fiscal policies would be relevant for countries in which those are being considered.

Lastly, we focused our analysis on European countries, which might not be representative of other regional contexts. The countries included in our analysis exhibited a broad spectrum of VAT rates on foods, including zero-rate, maximum-rate and reduced-rate schemes. We therefore think our results can provide some indication on likely trends in other regions with similar VAT systems, but exact impacts might differ depending on current food demand and prices, among other things. Although we would have liked to include additional regions in our analysis, we could not identify comparable datasets with VAT rates on food commodities. We would like to encourage the development of a global database to aid in extending this analysis.

## Methods

To analyse the health, environmental and cost implications of reforming VAT rates on foods in Europe, we first compiled a database of VAT rates on food products and aggregated them to general food groups. Second, we devised a set of policy scenarios in which VAT rates on F&V were reduced to zero and VAT rates on M&D were increased to the maximum rate in each country. Third, we used a new and economically consistent demand system to assess the impacts that changes in VAT rates would have on food demand. Fourth, we coupled the demand changes to a set of region- and commodity-specific footprints to assess the impacts of VAT reform on food-related GHG emissions, land use, freshwater use and eutrophication potential. Fifth, we translated the VAT-related changes in food demand into changes in intake of foods that are associated with non-communicable diseases (for example, low intake of vegetables and CHD), and calculated the changes in disease burden. Sixth, we coupled the changes in food demand to a set of country- and commodity-specific food prices to estimate changes in food budgets and tax revenues.

### VAT rates

We sourced data on VAT rates within Europe from the Taxes in Europe Database of the European Commission. This online database contains information on around 650 taxes based on information provided by the ministries of finance of the EU member states. The information is disaggregated to the Combined Nomenclature level, the European Union’s eight-digit coding system that comprises the Harmonized System codes, a standardized numerical method of classifying traded products, with further EU-specific subdivisions. We supplemented the dataset with data for the United Kingdom, which we extracted from government documents. For further analysis, we aggregated the food-related VAT rates from the Combined Nomenclature level (for example, lemons and limes) to the level of 24 general food groups (for example, fruits) (Supplementary Information Datafile).

### Demand analysis

We developed a comprehensive and economically consistent demand system to analyse how changes in VAT rates impact the demand of foods (Supplementary Section 1). The demand system resolves own-price effect governed by own-price elasticities, cross-price effects governed by cross-price elasticities, and income effects governed by the use of uncompensated elasticities of demand instead of compensated ones. To calibrate the demand system, we obtained country-specific food demand elasticities. We used a meta-analysis of food elasticities containing more than 50,000 food demand elasticities collected from 444 studies^28^ to train the supervised machine-learning algorithm XGBoost^27,53,54^. We then used data on the socioeconomic conditions of the countries covered in our analysis (for example, GDP per capita, urbanization and age characteristics) to obtain country-specific predictions. Finally, we used a maximum-entropy approach^27,55,56^ to calibrate the elasticities to comply with the theoretical conditions of consumer theory (Engel and Cournot aggregations), which ensures economic consistency.

### Health and environmental analyses

To analyse the health implications of VAT reform, we used estimates of food demand and the percentage of foods wasted^57,58^ to map the changes in food demand to changes in food intake, and we then used these changes in a comparative risk assessment of diet- and weight-related risks^29,30^ (Supplementary Section 2). The assessment included eight diet- and weight-related risk factors and five disease endpoints. The risk factors were high consumption of red meat, low consumption of fruits, vegetables, nuts and legumes, and being underweight, overweight or obese, the latter of which is related to changes in energy intake from all foods^3^. The disease endpoints were CHD, stroke, type 2 diabetes mellitus, cancer (in aggregate and as colorectal cancers), and respiratory disease. To parameterize the comparative risk assessment, we used data on cause-specific mortality, population and body weight^29–31^, and relative risk estimates that relate change in risk factors to changes in disease mortality^21–23,47–50^.

In the environmental analysis, we paired the VAT-related changes in food demand with a set of environmental footprints^6^, with endpoints including GHG emissions, freshwater use, land use and eutrophication potential (Supplementary Section 3). The footprints were based on a global meta-analysis of over 38,000 farms producing 40 different agricultural goods that have been regionalized for different world regions, including Europe.

### Cost analysis

In the cost analysis, we paired the changes in food demand with a set of country- and commodity-specific market prices of foods that were collected as part of the World Bank’s International Comparison Program to calculate differences in purchasing-power parity^34^. In previous research, we aggregated the International Comparison Program data (covering 463 food items with 20,666 estimates of annual average prices in 179 countries) to a list of 31 food groups commonly used to construct diet scenarios^33^. For the aggregation, we paired each item with its caloric content (sourced from US Department of Agriculture’s FoodData Central database) to control for differences in processing and edible fractions, and we converted averaged prices from local currency to international dollars based on purchasing-power parity rates that control for differences in price levels across countries (Supplementary Section 3).

As additional cost components, we included an analysis of changes in the costs to society that are not currently covered by market prices, including changes in healthcare-related costs and in the costs of climate damages (Supplementary Section 3). For the former, we used a set of country-specific cost-of-illness estimates covering both the direct cost (medical and healthcare costs) and the indirect costs (informal care and lost working days) associated with treating CHD, stroke, cancer and type 2 diabetes^3,4^. For the latter, we paired the estimated changes in GHG emissions with estimates of the social cost of carbon derived by the Greenhouse Gas Impact Value Estimator^38^. The social cost of carbon represents the economic cost caused by an additional tonne of GHG emissions.

### Reporting summary

Further information on research design is available in the Nature Portfolio Reporting Summary linked to this article.

## Supplementary information

Supplementary Information Supplementary Sections 1–4, Figs. 1–4 and Tables 1–9.

Reporting Summary

Supplementary Data Supplementary Datafile.

## Data Availability

All data produced in this study are available via Zenodo at 10.5281/zenodo.14057288 (ref. ^59^).

## Code availability

The codes for the trade and health analyses are described in detail in the Supplementary Information and the references cited therein.
