# Supplementary material for: A reform of value-added taxes on foods can have health, environmental and economic benefits in Europe
Source: Nat Food. 2025 Jan 9;6(2):161–9. doi: 10.1038/s43016-024-01097-5 (PMC11850278; doi:10.1038/s43016-024-01097-5)
Supplement: Supplementary file 1 — Supplementary Sections 1–4, Figs. 1–4 and Tables 1–9. [file 43016_2024_1097_MOESM1_ESM.pdf]

nature food

Supplementary information

https://doi.org/10.1038/s43016-024-01097-5

A reform of value-added taxes on foods can have health, environmental and economic benefits in Europe

In the format provided by the authors and unedited

Table of Contents

SI.1. Demand system 2

SI.2. Comparative risk assessment 6

SI.3. Environmental and cost analyses 9

SI.4. Supplementary results 10

References 18

SI.1. Demand system

To determine the changes in food demand consumption we use the demand system based of Robinson and colleagues[1],

$$FD'_{i,c} = FD^0_{i,c} \times \left(\frac{P'_{i,c}}{P^0_{i,c}}\right)^{\varepsilon_i} \prod_{j \neq i} \left(\frac{P'_{j,c}}{P^0_{j,c}}\right)^{\varepsilon_{ij}}$$

where we express the new food demand for food *i* in country *c* ($FD'_{i,c}$) in terms of the original food demand ($FD^0_{i,c}$), the change in the price of food i ($P'_{i,c}/P^0_{i,c}$), the uncompensated own-price elasticity (ε_i), the changes in prices in the rest of the goods ($P'_{j,c}/P^0_{j,c}$), and the uncompensated cross-price elasticities between food *i* and the rest of foods *j* (ε_{ij}).

To parameterize this demand system, we use Marshallian or uncompensated elasticities as they incorporate both the income (i.e., the effect derived from a change on real income due to the price change) and substitution effects from a price change.
The methodology we used to predict food demand elasticities is based on Bouyssou and colleagues[2] and summarized in SI Figure 1. We summarize its main aspects below.

SI Figure 1. Summary of the methodology for predicting food demand elasticities. Source: Adapted from ref.[2]

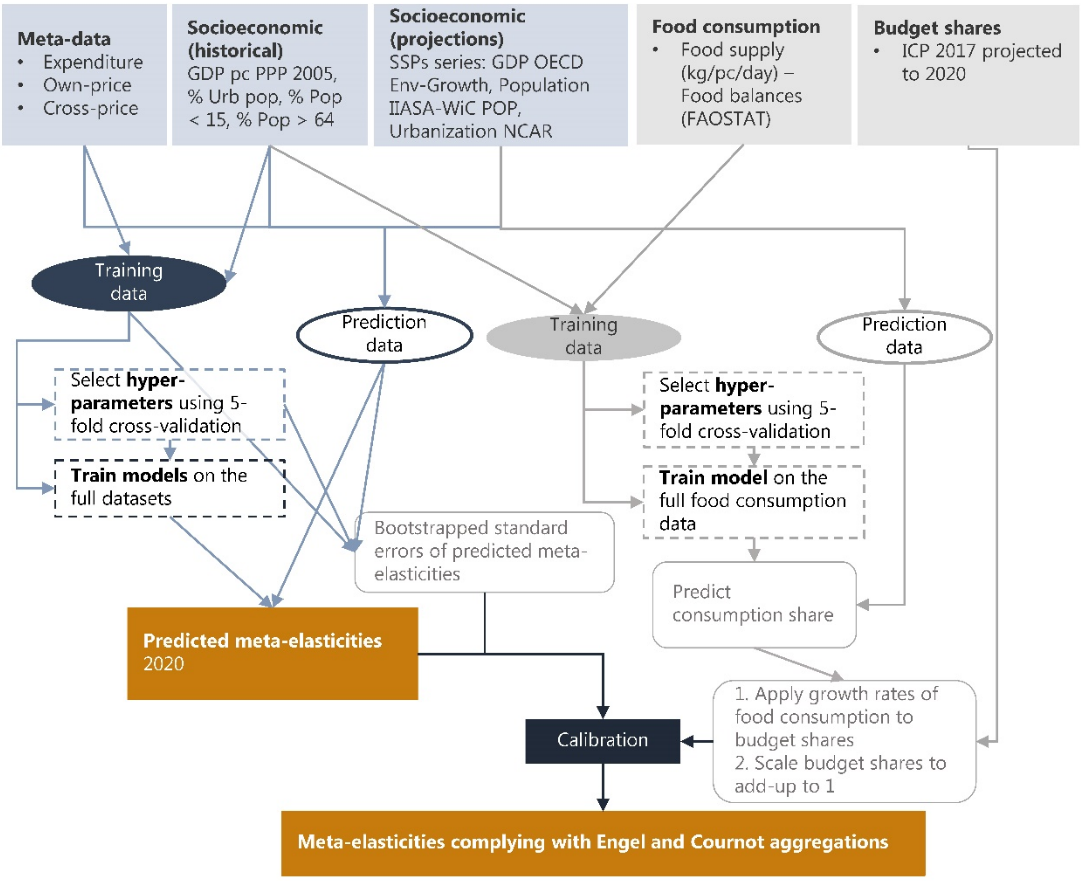

To predict food demand elasticities we used data collected for a meta-analysis containing more than 50,000 food demand elasticities collected from 444 studies in the literature [3]. In this case, instead of using an econometric model, we used a supervised machine learning

algorithm. The advantages are that (i) it is able to capture non-linear relationships, (ii) it accepts a larger number of interactions between country-level socio-economic variables and the different food groups, and (iii) it is in general better suited for out-of-sample prediction.

Among the many supervised machine learning algorithms, we considered ensemble regression trees the most appropriate to predict the elasticities[4]. Regression trees use dichotomous splits in the features to predict the outcome variable. For example, trying to predict income elasticities (ε_y), the algorithm could choose GDP > 10,000 INT$PPP to split the sample into two and predict $\hat{\varepsilon}_{y,GDP>10,000}$ income elasticities for the higher income observations and $\hat{\varepsilon}_{y,GDP\leq 10,000}$ for the remaining half. The trees are built by selecting splits that minimize prediction errors. Ensemble regression trees combine predictions from multiple decision trees, leading to more robust predictions. Moreover, in this class of algorithms, the importance that different features have on the prediction can be traced back.

Several regression tree algorithms exist, each with different properties. We chose to use XGBoost[5] for a number of reasons. First, XGBoost regression trees are built to iteratively improve predictions by minimizing the prediction errors from the previous trees using a gradient boosting algorithm. This makes it more efficient than algorithms using independent regression trees (e.g., Random Forests[6] and Extremely Randomized Trees[7]). Second, compared to other ensemble regression tree algorithms using gradient boosting (i.e., LightGBM[8] and CatBoost[9]), XGBoost is fast without compromising the accuracy of the predictions[10]. Finally, XGBoost is designed to prevent overfitting (i.e., learning from the noise in the training data), because it minimizes an objective function (the mean squared error (MSE) in our case) while simultaneously penalizing the complexity of the tree[5].

XGBoost predictions depend on the training data and the hyperparameters used. To train the algorithm we use a sample of 41,723 observations from 415 studies[1]. We separate the data into three smaller datasets, one for each type of elasticity (income, own-price, and cross-price). Then, we group the elasticities into 16 food groups: ruminants, pork, poultry, fresh dairy, preserved & other dairy, cheese, eggs, fats and oils, seafood, cereals, fruits, vegetables, pulses & nuts, tubers, sweets, and other food. SI Table 1 summarizes the number of observations by food group and elasticity type.

SI Table 1. Number of observations by food group and type of elasticity.

	Income	Own-price	Cross-price
Food	154	128	15
Animal food	4155	4755	14571
Meat	2270	2434	7806
Red meat	1079	1143	3404
Ruminants	523	591	2139
Pork	469	445	1139
White meat & eggs	772	842	2892
Poultry	506	561	1863
Eggs	260	271	974
Dairy	747	893	2684
Fresh dairy	299	389	1109
Preserved & other dairy	71	87	330
Cheese	102	104	401
Seafood	882	1154	3570

[1] From the sample we exclude beverages and other food groups not matching with the food groups used in this study.

Fats & oils	504	501	1687
Cereals	1166	1143	3693
Pulses & nuts	205	166	630
Tubers	115	98	549
Fruits & vegetables	1045	1039	3512
Fruits	401	386	1432
Vegetables	482	438	1466
Sweets	167	216	682
Other food	137	166	524
Total sample size	7648	8212	25863

Note: The shares of the disaggregated products do not add up to that for the aggregated group, this is to avoid losing the information if an elasticity is only available for an aggregated category (e.g., animal food, meat, or dairy) but not for a disaggregated product. We use all the observations to train the model even if after we predict only the elasticities for the groups indicated in italics. In the data we categorize the disaggregated groups (e.g., beef) in the respective aggregated group (i.e., red meat), and the more aggregated groups it belongs to (i.e., meat and animal food). The residual category is the generic Food, which captures elasticities estimated for food as a whole.

To include socioeconomic variables, we merged the datasets with World Bank[11] indicators: % urban population, % population under 15 years old, and % population over 64 years old, and the GDP per capita. Then we created interactions between the different food groups and socio-economic variables, and between the food groups and the world region the country belongs to. These interactions are useful to predict demand patterns specific to a socio-economic setting and culture. In the case of the cross-elasticities the interactions are created with respect to the food commodity reflecting the quantity change. We cannot force these interactions (or any other feature) into the model specification, as done in econometric models. Instead, we allowed the algorithm to learn from the features and interactions, so they will be included in the trained model only if they have enough predictive power.

To select the hyperparameters we used a grid-search with 5-fold cross-validation. For the grid we used (i) the learning rate which scales the contribution of each tree (η ranges from 0.01 to 0.28 in 0.03 increments), (ii) the maximum depth of each tree (max_depth ranges from 3 to 6 in increments of 1), (iii) the number of trees in the ensemble (nrounds = 1000 allowing for early stopping), and (iv) the $\ell 2$-regularization parameter which when larger leads to predicted values closer to zero (in the grid we use 1, 5, 10, 15 for λ). The remaining hyperparameters were set to default values.

In the 5-fold cross-validation the dataset was split into 5 random folds. The model was trained on 4 folds and the prediction accuracy was measured calculating the root-mean-square error (RMSE) in the remaining fold. The process was repeated until all the combinations are exhausted. Finally, we jointly minimized the average RMSE for the train and test datasets to choose the set of hyperparameters that lead to better out-of-sample prediction without over-fitting. The selected hyperparameters are summarized in SI Table 2.

SI Table 2. Hyper-parameters selected with cross-validation.

	Income	Own-price	Cross-price
Learning rate	0.04	0.25	0.22
Maximum depth	4	3	3
Number of trees	924	558	658
$\boldsymbol{\ell 2}$-regularization	5	5	15

Once the model is trained with the selected hyper-parameters, we used the socioeconomic variables to predict the elasticities for 2020.

Finally, we calibrated the predicted elasticities to comply with theoretical conditions of consumer theory (Engel and Cournot aggregations) using a minimum cross-entropy penalty framework[12,13]. This framework assumes that we have a prior probability distribution of elasticities with a set of mean values $\hat{\varepsilon}$ (where ε_{ij} is the price elasticity between goods i and j and ε_{iy} the income elasticity of i) and an associated set of standard deviations σ and budget shares w. The calibrated elasticities ε were determined by minimizing the penalty function (Z) (adapted from ref. [13] eq. 5),

$$\min Z = ½ \cdot \sum_i \sum_j \left(\left(1 - \frac{\varepsilon_{ij} - \hat{\varepsilon}_{ij}}{\sigma_{\varepsilon ij}}\right) \cdot \ln \left(1 - \frac{\varepsilon_{ij} - \hat{\varepsilon}_{ij}}{\sigma_{\varepsilon ij}}\right) + \left(1 + \frac{\varepsilon_{ij} - \hat{\varepsilon}_{ij}}{\sigma_{\varepsilon ij}}\right) \cdot \ln \left(1 + \frac{\varepsilon_{ij} - \hat{\varepsilon}_{ij}}{\sigma_{\varepsilon ij}}\right) \right)$$

Subject to the constraints,
$\sum_i w_i \cdot \varepsilon_{yi}(c) = 1$ (Engel aggregation)
$\sum_i w_i \cdot \varepsilon_{ji}(c) = -w_j$ (Cournot aggregation)

The cross-entropy is a measure of the additional information required to go from the prior distribution given by $(\hat{\varepsilon}, \sigma)$ to the distribution of the calibrated elasticities ε. Thus, we picked the distribution that meets the aggregation constraints while requiring the least additional information.

In the calibration, we used budget shares derived from combining estimates of food demand with the market prices of foods (see section SI.3) [14–16]. While calibrating the income meta-elasticities, we imposed upper boundaries for grains/flours and fruits/vegetables/pulses/ tubers and lower boundaries for meat products, cheese and eggs. The boundaries were determined as the region average from previous meta-regressions[3] plus or minus 0.1, depending on whether the commodity group was subject to an upper or lower bound, respectively.

SI.2. Comparative risk assessment

We estimated the mortality and disease burden attributable to dietary and weight-related risk factors by calculating population impact fractions (PIFs) which represent the proportions of disease cases that would be avoided when the risk exposure was changed from a baseline situation to a counterfactual situation. For calculating PIFs, we used the general formula[17–19]:

$$PIF = \frac{\int RR(x)P(x)dx - \int RR(x)P'(x)dx}{\int RR(x)P(x)dx}$$

where $RR(x)$ is the relative risk of disease for risk factor level x, $P(x)$ is the number of people in the population with risk factor level x in the baseline scenario, and $P'(x)$ is the number of people in the population with risk factor level x in the counterfactual scenario. We assumed that changes in relative risks follow a dose-response relationship,[18] and that PIFs combine multiplicatively, i.e. $PIF = 1 - \prod_i (1 - PIF_i)$ where the *i*'s denote independent risk factors.[18,20]

The number of avoided deaths due to the change in risk exposure of risk *i*, $\Delta deaths_i$, was calculated by multiplying the associated PIF by disease-specific death rates, *DR*, and by the number of people alive within a population, *P*:

$$\Delta deaths_i(r,s,a,d) = PIF_i(r,s,a,d) \cdot DR(r,s,a,d) \cdot P(r,s,a)$$

where PIFs are differentiated by region *r*, sex *s*, age group *a*, and disease/cause of death *d*; the death rates are differentiated by region, sex, age group, and disease; the population groups are differentiated by region, sex, and age group; and the change in the number of deaths is differentiated by region, sex, age group, and disease.

We used publicly available data sources to parameterize the comparative risk analysis. Mortality and population data were adopted from the Global Burden of Disease project.[21] Baseline data on the weight distribution in each country were adopted from a pooled analysis of population-based measurements undertaken by the NCD Risk Factor Collaboration.[22]

The relative risk estimates that relate the risk factors to the disease endpoints were adopted from meta-analyses of prospective cohort studies for dietary and weight-related risks.[23–29] In line with the meta-analyses, we included non-linear dose-response relationships for fruits, vegetables, and nuts and seeds, and assumed linear dose-response relationships for the remaining risk factors. As our analysis was primarily focused on mortality from chronic diseases, we focused on adults aged 20 year or older, and we adjusted the relative-risk estimates for attenuation with age based on a pooled analysis of cohort studies focussed on metabolic risk factors,[30] in line with other assessments.[19,31] SI Table 3 provides an overview of the relative-risk parameters used.

The selection of risk-disease associations used in the health analysis was supported by available criteria used to judge the certainty of evidence, such as the Bradford-Hill criteria used by the Nutrition and Chronic Diseases Expert Group (NutriCoDE),[31] the World-Cancer-Research-Fund criteria used by the Global Burden of Disease project,[32] as well as NutriGrade (SI Table 4).[33] The certainty of evidence supporting the associations of dietary risks and disease outcomes as used here were graded as moderate or high with NutriGrade,[26–28] and/or assessed as probable or convincing by the Nutrition and Chronic Diseases Expert Group,[31] and by the World Cancer Research.[34] The certainty of evidence grading in each case relates to the general relationship between a risk factor and a health outcome, and not to a specific relative-risk value.

SI Table 3. Relative risk parameters (mean and low and high values of 95% confidence intervals) for dietary risks and weight-related risks.

Food group	Endpoint	Unit	RR mean	RR low	RR high	Reference
Processed meat	CHD	50 g/d	1.27	1.09	1.49	Bechthold et al (2019)
	Stroke	50 g/d	1.17	1.02	1.34	Bechthold et al (2019)
	Colorectal cancer	50 g/d	1.17	1.10	1.23	Schwingshackl et al (2018)
	Type 2 diabetes	50 g/d	1.37	1.22	1.55	Schwingshackl et al (2017)
Red meat	CHD	100 g/d	1.15	1.08	1.23	Bechthold et al (2019)
	Stroke	100 g/d	1.12	1.06	1.17	Bechthold et al (2019)
	Colorectal cancer	100 g/d	1.12	1.06	1.19	Schwingshackl et al (2018)
	Type 2 diabetes	100 g/d	1.17	1.08	1.26	Schwingshackl et al (2017)
Fruits	CHD	100 g/d	0.95	0.92	0.99	Aune et al (2017)
	Stroke	100 g/d	0.77	0.70	0.84	Aune et al (2017)
	Cancer	100 g/d	0.94	0.91	0.97	Aune et al (2017)
Vegetables	CHD	100 g/d	0.84	0.80	0.88	Aune et al (2017)
	Cancer	100 g/d	0.93	0.91	0.95	Aune et al (2017)
Legumes	CHD	57 g/d	0.86	0.78	0.94	Afshin et al (2014)
Nuts	CHD	28 g/d	0.71	0.63	0.80	Aune et al (2016)
Whole grains	CHD	30 g/d	0.87	0.85	0.90	Aune et al (2016b)
	Cancer	30 g/d	0.95	0.93	0.97	Aune et al (2016b)
	Type 2 diabetes	30 g/d	0.65	0.61	0.70	Aune et al (2016b)
Underweight	CHD	15<BMI<18.5	1.17	1.09	1.24	Global BMI Collab (2016)
	Stroke	15<BMI<18.5	1.37	1.23	1.53	Global BMI Collab (2016)
	Cancer	15<BMI<18.5	1.10	1.05	1.16	Global BMI Collab (2016)
	Respiratory disease	15<BMI<18.5	2.73	2.31	3.23	Global BMI Collab (2016)
Overweight	CHD	25<BMI<30	1.34	1.32	1.35	Global BMI Collab (2016)
	Stroke	25<BMI<30	1.11	1.09	1.14	Global BMI Collab (2016)
	Cancer	25<BMI<30	1.10	1.09	1.12	Global BMI Collab (2016)
	Respiratory disease	25<BMI<30	0.90	0.87	0.94	Global BMI Collab (2016)
	Type 2 diabetes	25<BMI<30	1.88	1.56	2.11	Prosp Studies Collab (2009)
Obesity (grade 1)	CHD	30<BMI<35	2.02	1.91	2.13	Global BMI Collab (2016)
	Stroke	30<BMI<35	1.46	1.39	1.54	Global BMI Collab (2016)
	Cancer	30<BMI<35	1.31	1.28	1.34	Global BMI Collab (2016)
	Respiratory disease	30<BMI<35	1.16	1.08	1.24	Global BMI Collab (2016)
	Type 2 diabetes	30<BMI<35	3.53	2.43	4.45	Prosp Studies Collab (2009)
Obesity (grade 2)	CHD	30<BMI<35	2.81	2.63	3.01	Global BMI Collab (2016)
	Stroke	30<BMI<35	2.11	1.93	2.30	Global BMI Collab (2016)
	Cancer	30<BMI<35	1.57	1.50	1.63	Global BMI Collab (2016)
	Respiratory disease	30<BMI<35	1.79	1.60	1.99	Global BMI Collab (2016)
	Type 2 diabetes	30<BMI<35	6.64	3.80	9.39	Prosp Studies Collab (2009)
Obesity (grade 3)	CHD	30<BMI<35	3.81	3.47	4.17	Global BMI Collab (2016)
	Stroke	30<BMI<35	2.33	2.05	2.65	Global BMI Collab (2016)
	Cancer	30<BMI<35	1.96	1.83	2.09	Global BMI Collab (2016)
	Respiratory disease	30<BMI<35	2.85	2.43	3.34	Global BMI Collab (2016)
	Type 2 diabetes	30<BMI<35	12.49	5.92	19.82	Prosp Studies Collab (2009)

We did not include all available risk-disease associations that were graded as having a moderate certainty of evidence and showed statistically significant results in the meta-analyses that included NutriGrade assessments.[26–28] That was because for some associations, such as for milk and fish, more detailed meta-analyses (with more sensitivity analyses) were available that indicated potential confounding with other major dietary risks or health status at baseline.[35–37] Such sensitivity analyses were not presented in the meta-

analyses that included NutriGrade assessments, but they are important for health assessments that evaluate changes in multiple risk factors.

SI Table 4. Overview of existing ratings on the certainty of evidence for a statistically significant association between a risk factor and a disease endpoint. The ratings include those of the Nutrition and Chronic Diseases Expert Group (NutriCoDE),[31] the World Cancer Research Fund,[34] and NutriGrade.[26–28] The ratings relate to the risk-disease associations in general, and not to the specific relative-risk factor used for those associations in this analysis.

Food group	Endpoint	Association	Certainty of evidence
Fruits	CHD	reduction	NutriCoDE: probable or convincing;<br>NutriGrade: moderate quality of meta-evidence
	Stroke	reduction	NutriCoDE: probable or convincing<br>NutriGrade: moderate quality of meta-evidence
	Cancer	reduction	WCRF: strong evidence (probable) for some cancers<br>NutriGrade: moderate quality of meta-evidence for colorectal cancer
Vegetables	CHD	reduction	NutriCoDE: probable or convincing<br>NutriGrade: moderate quality of meta-evidence
	Cancer	reduction	WCRF: strong evidence (probable) for non-starchy vegetables and some cancers<br>NutriGrade: moderate quality of meta-evidence for colorectal cancer
Legumes	CHD	reduction	NutriCoDE: probable or convincing<br>NutriGrade: moderate quality of meta-evidence
Nuts and seeds	CHD	reduction	NutriCoDE: probable or convincing<br>NutriGrade: moderate quality of meta-evidence
Whole grains	CHD	reduction	NutriCoDE: probable or convincing<br>NutriGrade: moderate quality of meta-evidence
	Cancer	reduction	WCRF: strong evidence (probable) for colorectal cancer<br>NutriGrade: moderate quality of meta-evidence for colorectal cancer
	Type-2 diabetes	reduction	NutriCoDE: probable or convincing<br>NutriGrade: high quality of meta-evidence
Red meat	CHD	increase	NutriGrade: moderate quality of meta-evidence
	Stroke	increase	NutriGrade: moderate quality of meta-evidence
	Cancer	increase	WCRF: strong evidence (probable) for colorectal cancer<br>NutriGrade: moderate quality of meta-evidence for colorectal cancer
	Type-2 diabetes	increase	NutriCoDE: probable or convincing<br>NutriGrade: high quality of meta-evidence
Processed meat	CHD	increase	NutriCoDE: probable or convincing<br>NutriGrade: moderate quality of meta-evidence
	Stroke	increase	NutriGrade: moderate quality of meta-evidence
	Cancer	increase	WCRF: strong evidence (convincing) for colorectal cancer<br>NutriGrade: moderate quality of meta-evidence for colorectal cancer
	Type-2 diabetes	increase	NutriGrade: high quality of meta-evidence

NutriCoDE: Nutrition and Chronic Diseases Expert Group
NutriGrade: Grading of Recommendations Assessment, Dvelopment, and Evaluation (GRADE) tailored to nutrition research
WCRF: World Cancer Research Fund

For the different diet scenarios, we calculated uncertainty intervals associated with changes in mortality based on standard methods of error propagation and the confidence intervals of the relative risk parameters. For the error propagation, we approximated the error distribution of the relative risks by a normal distribution and used that side of deviations from the mean which was largest. This method leads to conservative and potentially larger uncertainty intervals as probabilistic methods, such as Monte Carlo sampling, but it has significant computational advantages, and is justified for the magnitude of errors dealt with here (<50%) (see e.g. IPCC Uncertainty Guidelines).

SI.3. Environmental and cost analyses

We assessed the environmental impacts of VAT reform by using a set of region-specific environmental footprints, specifying the GHG emissions, land use, freshwater use, and eutrophication potential of foods that accrue throughout their lifecycle, including production, inputs, and transport to the point of consumption [38]. The footprints were adapted from a meta-analysis of 570 life-cycle assessments covering results from over 38,000 farms in 119 countries. For the assessment, we paired the environmental footprints with food demand estimated in the different scenarios of VAT reform and with baseline demand.

We assessed the cost implications of VAT reform by estimating changes in the cost of diets at the level of food purchases. For the assessment, we paired the estimates of food demand in the different scenarios of VAT reform with data on food prices that were collected by statistical offices for the year 2017 as part of the International Comparison Program (ICP) led by the World Bank.[15] We used a total of 20,666 estimates of annual average prices in 179 countries, covering 463 food items, which we aggregated to a list of 31 food groups that match the scenarios on meal composition.[16] For the aggregation, we paired each item with its caloric content (to control for difference in processing and edible fractions), and converted averaged prices from local currency to USD using purchasing power parity rates, which controls for differences in price levels across countries.

We also assessed some of the costs associated with foods but that are currently external to food prices. They included the costs of climate-change damages that are associated with food-related GHG emissions, and the cost of illness associated with dietary and weight-related risk factors. For estimating the cost of climate-change damages, we used estimates of the social cost of carbon (SCC), representing the monetised value of the damages to society caused by an incremental metric tonne of carbon dioxide emissions, from the Greenhouse Gas Impact Value Estimator (GIVE), a probabilistic integrated assessment model with updated systems components.[39] The suggested SCC value we adopted was US$ 185 per tCO2 (95% CI, US$ 44-413) for a 2% near-term discount rate.

For estimating the health-related costs of dietary and weight-related diseases, we used estimates of the cost of illness associated with coronary heart disease, stroke, type-2 diabetes, and cancer. Cost-of-illness estimates capture both the direct and indirect costs associated with treating a specific disease, including medical and health-care costs (direct), and costs of informal care and from lost working days.[40] For our calculations, we used a global set of country-specific cost-of-illness estimates adopted from Springmann and colleagues.[41]

SI.4. Supplementary results

SI Table 5. VAT rates on food groups and categories in Europe. Min and max rates denote the minimum and maximum rates across all foods and within each food group.

Region	Category		Food group									All foods	
	meat &dairy	fruits &veg	beef	lamb	pork	poultry	milk	fruits	veg	legume	nuts	min rate	max rate
Europe	8.4	9.3	8.0	8.3	8.0	7.8	8.7	9.7	8.8	9.0	9.3	7.2	21.0
Eastern Europe	8.6	11.0	9.8	10.4	9.5	8.7	8.3	11.3	10.9	11.5	11.1	10.2	21.9
Northern Europe	5.0	5.6	5.4	5.8	5.8	5.8	4.8	5.7	5.5	6.5	5.7	5.7	21.2
Southern Europe	13.5	13.2	10.6	10.8	10.6	10.2	15.2	13.7	12.4	12.5	14.2	7.0	22.0
Western Europe	6.5	7.6	6.7	6.7	6.7	6.7	6.4	8.3	7.1	6.7	6.7	6.7	19.7
Austria	10.0	11.6	10.0	10.0	10.0	10.0	10.0	12.9	10.7	10.2	10.1	10.0	20.0
Belgium	2.2	6.1	6.0	6.0	6.0	6.0	0.2	6.3	6.0	6.0	6.0	6.0	21.0
Bulgaria	20.0	19.9	20.0	20.0	20.0	20.0	20.0	19.9	19.9	20.0	20.0	20.0	20.0
Cyprus	6.0	9.6	8.3	6.0	8.7	7.7	5.0	10.8	8.8	6.9	6.7	5.0	19.0
Czechia	12.0	12.0	12.0	12.0	12.0	12.0	12.0	12.0	12.0	12.0	12.0	12.0	21.0
Germany	7.0	8.7	7.0	7.0	7.0	7.0	7.0	10.1	7.5	7.0	7.0	7.0	19.0
Denmark	25.0	25.0	25.0	25.0	25.0	25.0	25.0	25.0	25.0	25.0	25.0	25.0	25.0
Spain	16.4	16.2	10.0	10.0	10.0	10.0	20.7	16.8	15.7	17.2	15.0	10.0	21.0
Estonia	22.0	22.0	22.0	22.0	22.0	22.0	22.0	22.0	22.0	22.0	22.0	22.0	22.0
Finland	14.0	14.1	14.0	14.0	14.0	14.0	14.0	14.3	14.0	14.0	14.2	14.0	24.0
France	5.5	5.6	5.5	5.5	5.5	5.5	5.5	5.5	5.7	5.5	5.5	5.5	20.0
United Kingdom	0.0	0.4	0.0	0.0	0.0	0.0	0.0	0.0	0.8	1.7	0.0	0.0	20.0
Greece	13.0	13.2	13.0	13.0	13.0	13.0	13.0	13.3	13.0	12.0	13.2	13.0	24.0
Croatia	9.5	13.6	14.4	16.4	14.3	12.6	7.2	8.3	14.9	9.7	8.7	5.0	25.0
Hungary	7.9	27.0	16.7	21.5	15.3	9.2	5.4	27.0	27.0	27.0	27.0	18.0	27.0
Ireland	9.0	14.9	14.9	23.0	23.0	23.0	5.0	21.9	7.6	13.2	22.2	23.0	23.0
Italy	12.7	11.4	10.0	10.0	10.0	10.0	13.7	12.5	9.9	9.2	14.1	4.0	22.0
Lithuania	21.0	21.0	21.0	21.0	21.0	21.0	21.0	21.0	21.0	21.0	21.0	21.0	21.0
Luxembourg	3.0	3.6	3.0	3.0	3.0	3.0	3.0	3.5	3.6	3.6	3.0	3.0	17.0
Latvia	13.9	12.1	18.9	20.4	18.6	19.3	12.0	12.1	12.1	12.3	12.0	12.0	21.0
Malta	1.8	1.8	4.2	1.3	4.8	3.4	0.0	4.7	0.2	0.0	0.8	0.0	18.0
Netherlands	9.0	9.0	9.0	9.0	9.0	9.0	9.0	9.0	9.0	9.0	9.0	9.0	21.0
Poland	5.0	5.2	5.0	5.6	5.0	5.0	5.0	5.5	5.0	5.8	5.1	5.0	23.0
Portugal	8.6	11.3	13.0	15.1	12.8	9.2	6.5	10.4	11.8	13.5	16.1	6.0	23.0
Romania	9.0	9.1	9.0	9.0	9.0	9.0	9.0	9.3	9.0	9.0	9.0	9.0	19.0
Slovakia	12.8	16.5	16.5	13.6	14.7	11.9	12.2	17.5	15.5	20.0	19.8	20.0	20.0
Slovenia	9.5	9.7	9.5	9.5	9.5	9.5	9.5	10.0	9.5	9.5	9.5	9.5	22.0
Sweden	12.0	12.4	12.0	12.0	12.0	12.0	12.0	12.4	12.4	14.0	12.4	12.0	25.0

SI Figure 2. VAT rates on meat and dairy (a) and on fruits and vegetables (b) across Europe.

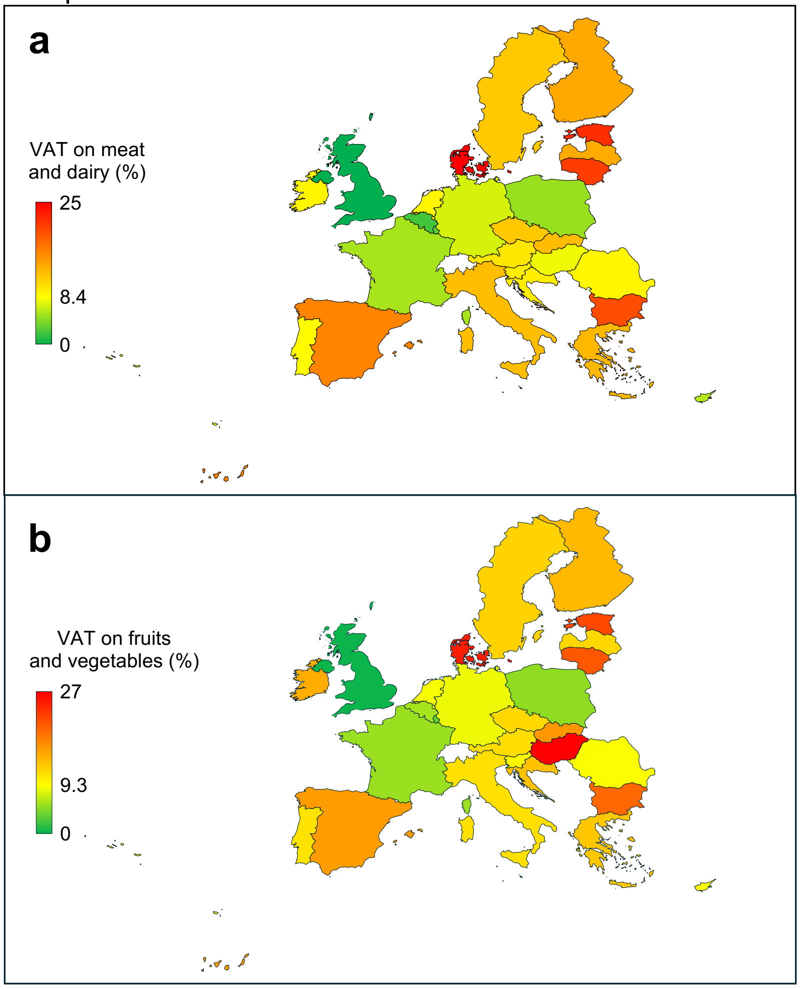

SI Figure 3. Change in the demand for meat and dairy (M&D) and fruits and vegetables (F&V) for separate changes in VAT rates on these food categories (**a, b**) and for combined changes (**c, d**).

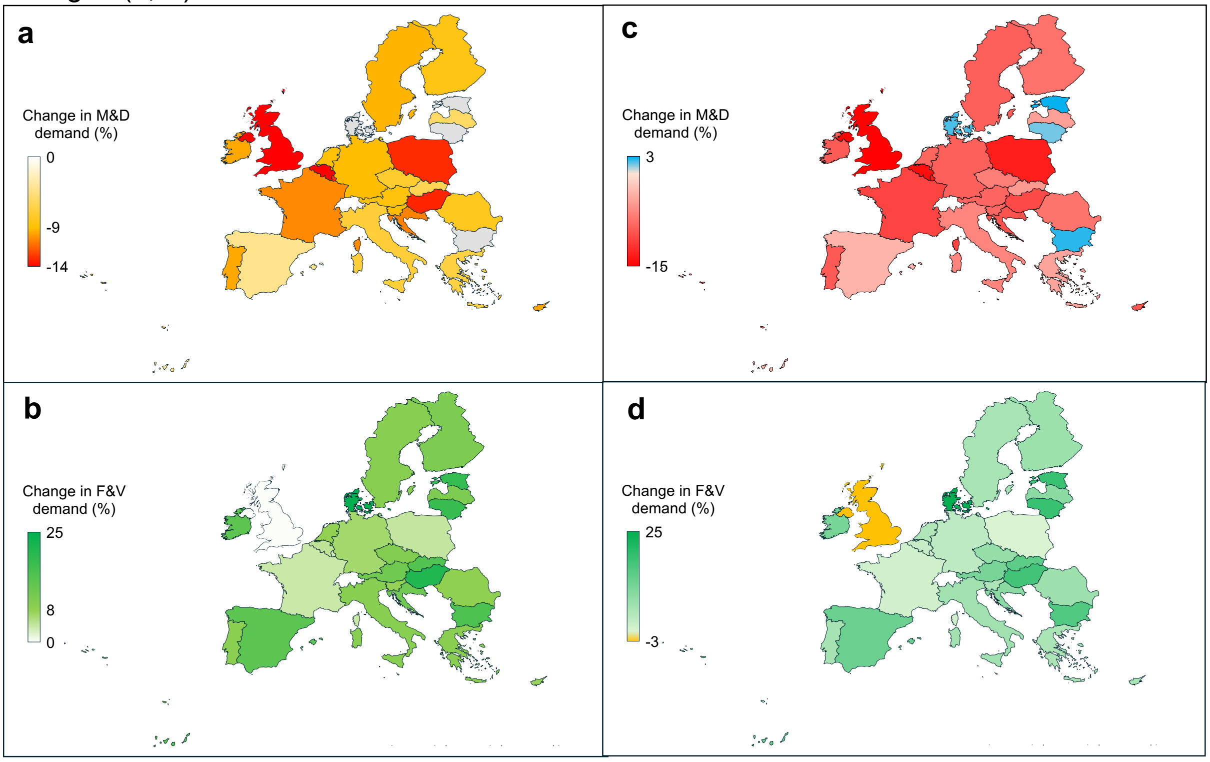

SI Table 6. Change in environmental resource use and pollution for combined changes in the VAT rates on foods. The environmental endpoints include GHG emissions ($MtCO_2eq$), land use (Land, thousand km^2), freshwater use (Water, km^3), and eutrophication potential ($PO^4{}_3$-eq). The average percentage changes across all environmental endpoints is denoted as Average.

Region	Change in environmental impacts (%)					Absolute change in environmental impacts			
	Average	GHG	Land	Water	Eutr	GHG	Land	Water	Eutr
Europe	-5.7	-6.2	-5.6	-5.3	-5.6	-63.3	-71.3	-8,240.3	-208.4
Eastern Europe	-5.5	-5.7	-5.0	-6.1	-5.1	-8.4	-8.8	-1,465.7	-27.5
Northern Europe	-8.6	-8.8	-8.7	-8.3	-8.7	-18.4	-22.7	-2,259.6	-61.1
Southern Europe	-3.0	-3.8	-3.0	-2.0	-3.2	-10.7	-11.4	-950.6	-36.6
Western Europe	-6.4	-6.7	-6.2	-6.3	-6.2	-25.8	-28.4	-3,564.4	-83.2
Austria	-4.1	-4.9	-4.0	-3.6	-3.8	-0.9	-0.8	-99.1	-2.7
Belgium	-7.3	-7.7	-7.1	-7.4	-7.2	-1.7	-2.0	-250.8	-5.3
Bulgaria	3.1	2.8	3.1	3.8	2.6	0.3	0.4	55.3	1.0
Cyprus	-5.2	-5.9	-5.0	-4.8	-4.9	-0.1	-0.1	-18.9	-0.4
Czechia	-3.7	-3.9	-3.6	-3.9	-3.5	-0.8	-0.8	-105.9	-2.5
Germany	-5.7	-6.0	-5.4	-5.9	-5.6	-9.7	-10.0	-1,457.8	-31.4
Denmark	2.7	2.3	2.6	3.8	2.2	0.3	0.3	62.7	0.8
Spain	-2.1	-3.0	-2.4	-0.8	-2.3	-3.1	-3.4	-133.6	-10.1
Estonia	2.9	2.8	2.7	3.4	2.5	0.1	0.1	13.7	0.2
Finland	-5.0	-5.1	-4.7	-5.4	-4.9	-0.6	-0.6	-87.3	-1.7
France	-7.5	-7.8	-7.5	-7.5	-7.2	-11.4	-13.5	-1,452.4	-37.2
United Kingdom	-11.3	-11.5	-11.2	-11.3	-11.2	-16.4	-20.7	-2,022.6	-54.8
Greece	-1.7	-2.2	-1.7	-1.1	-1.9	-0.5	-0.5	-40.8	-1.8
Croatia	-3.9	-4.3	-3.4	-4.1	-3.9	-0.3	-0.3	-47.9	-1.1
Hungary	-4.4	-4.8	-3.6	-5.1	-4.0	-0.7	-0.7	-137.8	-2.3
Ireland	-3.9	-4.3	-3.3	-4.2	-3.6	-0.5	-0.5	-66.9	-1.2
Italy	-3.5	-4.3	-3.3	-2.7	-3.8	-5.3	-5.3	-557.9	-18.8
Lithuania	2.0	1.9	1.8	2.5	1.7	0.1	0.1	19.7	0.3
Luxembourg	-7.6	-7.6	-7.3	-7.7	-7.7	-0.1	-0.1	-10.2	-0.4
Latvia	-1.4	-1.5	-1.1	-1.9	-1.1	-0.1	0.0	-9.0	-0.1
Malta	-7.5	-7.9	-7.3	-7.4	-7.3	-0.1	-0.1	-7.8	-0.2
Netherlands	-5.2	-5.7	-4.9	-5.1	-5.2	-2.0	-1.9	-294.1	-6.2
Poland	-9.4	-9.1	-8.7	-10.2	-9.4	-5.6	-6.1	-1,044.5	-18.5
Portugal	-4.5	-5.1	-4.6	-3.6	-4.5	-1.1	-1.4	-136.7	-4.0
Romania	-3.6	-4.0	-3.6	-3.6	-3.3	-1.4	-1.5	-213.6	-4.8
Slovakia	-1.4	-1.7	-1.0	-1.6	-1.4	-0.1	-0.1	-19.2	-0.4
Slovenia	-4.9	-5.4	-4.8	-4.3	-4.8	-0.2	-0.2	-25.9	-0.7
Sweden	-6.0	-6.1	-5.8	-6.2	-6.0	-1.3	-1.5	-170.0	-4.5

SI Table 7. Number of averted deaths by cause of death and region in the scenario of combined VAT changes in fruits and vegetables and on meat and dairy. The causes of death include coronary heart disease (CHD), stroke, cancer, type-2 diabetes (T2DM), and respiratory disease (Resp Dis).

Region	Number of averted deaths					
	Total	CHD	Stroke	Cancer	T2DM	Resp Dis
Europe	167,408	91,109	24,373	47,479	3,891	556
Eastern Europe	43,188	27,187	6,372	9,122	487	20
Northern Europe	16,444	9,842	1,870	3,955	449	329
Southern Europe	67,818	31,540	12,009	23,336	922	11
Western Europe	39,958	22,541	4,123	11,066	2,033	196
Austria	4,762	2,960	351	1,380	71	0
Belgium	2,280	1,224	229	725	86	16
Bulgaria	5,782	4,071	782	1,022	-76	-18
Cyprus	166	87	23	39	17	1
Czechia	3,743	2,361	481	834	63	4
Germany	20,153	12,435	1,861	4,864	897	95
Denmark	3,832	1,573	358	1,953	-43	-10
Spain	24,571	10,377	4,266	9,741	209	-23
Estonia	1,021	656	133	237	-5	-1
Finland	1,764	1,052	230	458	18	6
France	9,116	4,495	1,068	2,619	866	67
United Kingdom	2,118	1,829	219	-637	384	323
Greece	7,606	4,111	1,451	2,033	11	1
Croatia	5,730	3,742	263	1,686	36	3
Hungary	9,064	5,196	1,209	2,608	47	5
Ireland	1,070	395	253	414	6	2
Italy	24,397	11,012	4,830	8,022	512	21
Lithuania	2,638	1,877	238	532	-5	-3
Luxembourg	72	39	12	16	4	1
Latvia	1,218	820	159	238	1	0
Malta	22	11	7	-3	6	1
Netherlands	3,575	1,388	601	1,461	108	16
Poland	7,644	5,644	509	1,053	412	26
Portugal	4,826	1,959	1,099	1,621	139	8
Romania	14,590	8,407	3,102	3,036	42	3
Slovakia	2,365	1,507	289	569	0	0
Slovenia	665	329	92	236	9	0
Sweden	2,783	1,639	280	760	93	11

SI Table 8. Comparison between the scenario of combined VAT reform on reducing rates on fruits and vegetables and increasing rates on meat and dairy (VAT) with one in which rates are only increased for meat (MEA) and one in which rates are only increased on red meat (RDM), whilst maintaining the reduction in VAT rates on fruits and vegetables in both cases.

Region	Averted deaths (per 1000 people)			Change in environmental impacts (%)			Change in VAT revenues (% GDP)		
	VAT	MEA	RDM	VAT	MEA	RDM	VAT	MEA	RDM
Europe	0.33	0.34	0.34	-5.65	-3.41	-2.56	0.22	0.13	0.10
Eastern Europe	0.48	0.48	0.48	-5.46	-2.91	-1.70	0.28	0.16	0.11
Northern Europe	0.17	0.17	0.17	-8.60	-5.59	-4.35	0.35	0.26	0.22
Southern Europe	0.51	0.53	0.53	-2.97	-1.93	-1.24	0.04	0.00	-0.03
Western Europe	0.21	0.22	0.22	-6.36	-3.67	-3.02	0.22	0.13	0.11
Austria	0.54	0.54	0.55	-4.06	-2.31	-1.96	0.04	-0.01	-0.02
Belgium	0.20	0.22	0.23	-7.32	-4.04	-3.47	0.26	0.08	0.06
Bulgaria	0.82	0.82	0.82	3.07	3.07	3.07	-0.39	-0.39	-0.39
Cyprus	0.18	0.16	0.15	-5.15	-2.57	-1.69	0.16	0.06	0.03
Czechia	0.35	0.35	0.34	-3.74	-1.98	-1.31	0.07	0.00	-0.02
Germany	0.24	0.26	0.26	-5.71	-3.00	-2.35	0.17	0.08	0.06
Denmark	0.66	0.66	0.66	2.73	2.73	2.73	-0.26	-0.26	-0.26
Spain	0.53	0.53	0.52	-2.14	-2.10	-1.31	-0.02	-0.02	-0.06
Estonia	0.78	0.78	0.78	2.86	2.86	2.86	-0.41	-0.41	-0.41
Finland	0.32	0.31	0.31	-5.01	-2.39	-1.73	0.16	0.09	0.07
France	0.14	0.14	0.12	-7.50	-4.81	-4.04	0.35	0.26	0.23
United Kingdom	0.03	0.03	0.04	-11.28	-7.79	-6.11	0.57	0.45	0.39
Greece	0.71	0.83	0.84	-1.72	0.43	0.97	-0.10	-0.21	-0.25
Croatia	1.39	1.49	1.48	-3.88	-1.11	-0.51	0.02	-0.13	-0.16
Hungary	0.93	0.95	0.93	-4.37	-0.53	1.12	0.00	-0.14	-0.22
Ireland	0.22	0.25	0.25	-3.87	-0.19	-0.19	0.01	-0.06	-0.06
Italy	0.41	0.43	0.43	-3.50	-2.10	-1.53	0.08	0.03	0.01
Lithuania	0.93	0.93	0.93	1.97	1.97	1.97	-0.36	-0.36	-0.36
Luxembourg	0.12	0.12	0.10	-7.56	-6.01	-5.14	0.19	0.15	0.15
Latvia	0.64	0.64	0.63	-1.40	0.74	0.91	-0.13	-0.21	-0.22
Malta	0.05	0.07	0.07	-7.48	-5.25	-4.20	0.33	0.22	0.19
Netherlands	0.21	0.23	0.23	-5.24	-2.45	-2.09	0.13	0.05	0.04
Poland	0.20	0.21	0.20	-9.35	-6.16	-4.22	0.66	0.51	0.43
Portugal	0.47	0.51	0.50	-4.47	-2.56	-1.64	0.14	0.04	-0.01
Romania	0.77	0.77	0.79	-3.62	-1.39	-0.80	0.07	-0.04	-0.06
Slovakia	0.43	0.43	0.42	-1.44	0.05	0.62	-0.11	-0.15	-0.17
Slovenia	0.32	0.36	0.35	-4.85	-2.74	-1.62	0.09	0.02	-0.01
Sweden	0.27	0.25	0.27	-6.01	-3.33	-2.85	0.15	0.07	0.05

SI Table 9. Comparison between the scenario of combined VAT reform with taxing foods according to their GHG emissions valued either with a social cost of carbon (SCC) of 185 USD/tCO_2eq (SCC) or with a carbon price that matched the emissions reductions of the VAT scenario (63 USD/tCO_2eq).

Region	Averted deaths			Change in GHG (%)		
	VAT	SCC	SCC-m	VAT	SCC	SCC-m
Europe	167,408	144,802	57,723	-6.17	-15.74	-6.19
Austria	4,762	2,796	1,113	-4.91	-17.60	-7.07
Belgium	2,280	2,621	903	-7.68	-15.96	-6.36
Bulgaria	5,782	4,162	2,019	2.81	-14.35	-5.60
Cyprus	166	194	73	-5.90	-14.24	-5.47
Czechia	3,743	3,737	1,436	-3.92	-15.35	-6.05
Germany	20,153	24,206	11,052	-6.00	-14.89	-5.79
Denmark	3,832	1,870	882	2.30	-18.08	-7.20
Spain	24,571	11,436	4,579	-3.04	-16.58	-6.63
Estonia	1,021	509	201	2.75	-14.67	-5.68
Finland	1,764	1,682	568	-5.05	-14.46	-5.61
France	9,116	16,708	6,386	-7.84	-16.91	-6.71
United Kingdom	2,118	10,947	4,079	-11.47	-17.68	-7.02
Greece	7,606	4,018	782	-2.20	-16.80	-6.65
Croatia	5,730	1,878	428	-4.25	-12.70	-4.86
Hungary	9,064	3,228	1,212	-4.80	-13.77	-5.32
Ireland	1,070	591	217	-4.31	-14.52	-5.60
Italy	24,397	27,234	11,571	-4.29	-15.17	-5.90
Lithuania	2,638	1,602	607	1.93	-14.80	-5.78
Luxembourg	72	103	55	-7.62	-13.37	-5.20
Latvia	1,218	979	381	-1.52	-15.13	-5.89
Malta	22	116	44	-7.91	-16.41	-6.47
Netherlands	3,575	4,049	1,293	-5.68	-14.19	-5.46
Poland	7,644	7,657	2,831	-9.13	-12.11	-4.63
Portugal	4,826	2,383	939	-5.11	-14.53	-5.66
Romania	14,590	5,259	1,910	-4.04	-14.44	-5.58
Slovakia	2,365	1,665	661	-1.69	-16.16	-6.43
Slovenia	665	303	117	-5.44	-16.37	-6.49
Sweden	2,783	2,868	1,383	-6.07	-16.48	-6.50

SI Figure 4. Comparison between changes in VAT revenues (**a**) and net benefits (**b**) that include changes in VAT revenues and reductions in healthcare-related costs and climate-change damages.

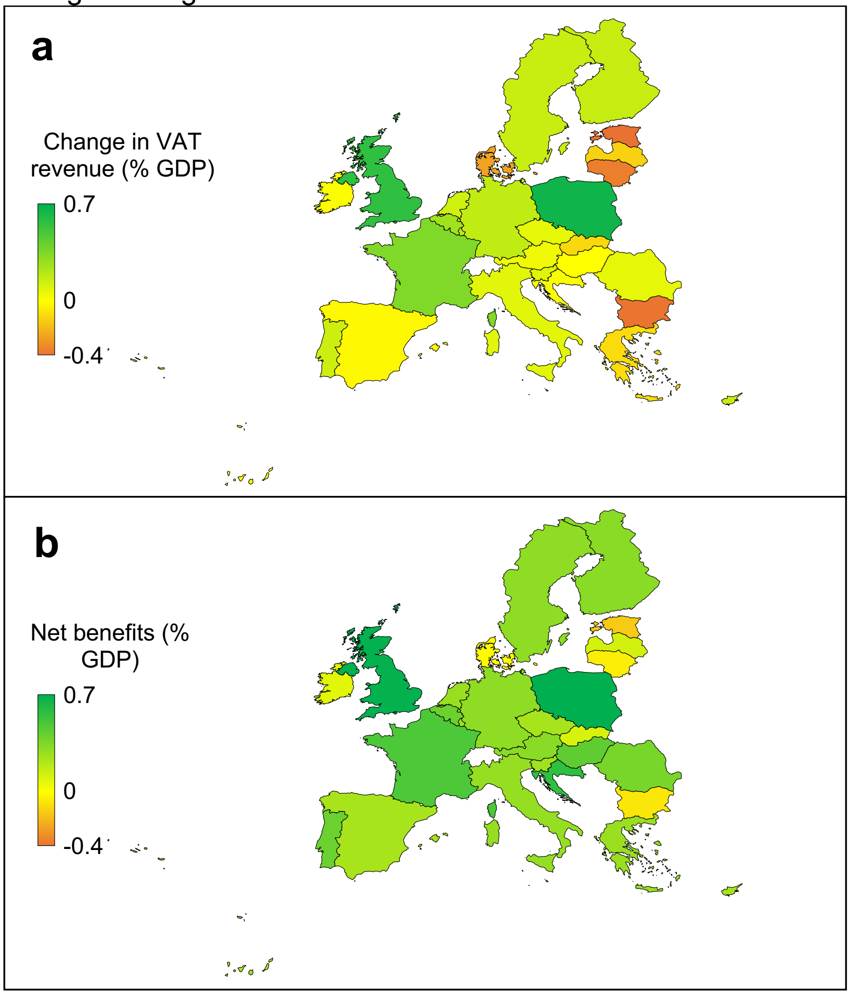

References

1. Robinson, S. et al. The International Model for Policy Analysis of Agricultural Commodities and Trade (IMPACT) – Model Description for Version 3. (International Food Policy Research Institute, 2015).
2. Bouyssou, C. G., Clora, F., Jensen, J. D. & Yu, W. A taste of tomorrow: Predicting food demand elasticities under different Shared Socioeconomic Pathways. In *The role of income, demographic, and incentive changes in future food demand scenarios: A pluralistic modeling approach* 73–106 (2024).
3. Bouyssou, C. G., Jensen, J. D. & Yu, W. Food for thought: A meta-analysis of animal food demand elasticities across world regions. Food Policy **122**, (2024).
4. Crisci, C., Ghattas, B. & Perera, G. A review of supervised machine learning algorithms and their applications to ecological data. Ecological Modelling **240**, 113–122 (2012).
5. Chen, T. & Guestrin, C. XGBoost: A Scalable Tree Boosting System. in Proceedings of the 22nd ACM SIGKDD International Conference on Knowledge Discovery and Data Mining 785–794 (Association for Computing Machinery, New York, NY, USA, 2016). doi:10.1145/2939672.2939785.
6. Breiman, L. Random Forests. Machine Learning **45**, 5–32 (2001).
7. Geurts, P., Ernst, D. & Wehenkel, L. Extremely randomized trees. Mach Learn **63**, 3–42 (2006).
8. Ke, G. et al. LightGBM: A Highly Efficient Gradient Boosting Decision Tree. in Advances in Neural Information Processing Systems vol. 30 (Curran Associates, Inc., 2017).
9. Prokhorenkova, L., Gusev, G., Vorobev, A., Dorogush, A. V. & Gulin, A. CatBoost: unbiased boosting with categorical features. in Advances in Neural Information Processing Systems vol. 31 (Curran Associates, Inc., 2018).
10. Bentéjac, C., Csörgő, A. & Martínez-Muñoz, G. A comparative analysis of gradient boosting algorithms. Artif Intell Rev **54**, 1937–1967 (2021).
11. World Bank. World Bank Indicators. https://data.worldbank.org/ (2022).
12. Golan, A., Judge, G., G. & Miller, D. Maximum Entropy Econometrics: Robust Estimation with Limited Data. (John Wiley & Sons, New York, 1996).
13. Preckel, P. V. Least Squares and Entropy: A Penalty Function Perspective. American Journal of Agricultural Economics **83**, 366–377 (2001).
14. Food and Agriculture Organization of the United Nations. FAOSTAT Statistical Database. (2022).
15. World Bank. Purchasing Power Parities and the Real Size of World Economies: A Comprehensive Report of the 2011 International Comparison Program. (2015) doi:doi:10.1596/978-1-4648-0329-1.
16. Springmann, M., Clark, M. A., Rayner, M., Scarborough, P. & Webb, P. The global and regional costs of healthy and sustainable dietary patterns: a modelling study. The Lancet Planetary Health **5**, e797–e807 (2021).
17. Murray, C. J. L., Ezzati, M., Lopez, A. D., Rodgers, A. & Vander Hoorn, S. Comparative quantification of health risks: conceptual framework and methodological issues. Population Health Metrics **1**, 1 (2003).
18. Lim, S. S. et al. A comparative risk assessment of burden of disease and injury attributable to 67 risk factors and risk factor clusters in 21 regions, 1990–2010: a systematic analysis for the Global Burden of Disease Study 2010. The Lancet **380**, 2224–2260 (2012).
19. Forouzanfar, M. H. et al. Global, regional, and national comparative risk assessment of 79 behavioural, environmental and occupational, and metabolic risks or clusters of risks in 188 countries, 1990–2013: a systematic analysis for the Global Burden of Disease Study 2013. The Lancet **386**, 2287–2323 (2015).
20. Murray, C. J. L. et al. GBD 2010: design, definitions, and metrics. Lancet **380**, 2063–2066 (2012).

21. Wang, H. et al. Global age-sex-specific fertility, mortality, healthy life expectancy (HALE), and population estimates in 204 countries and territories, 1950–2019: a comprehensive demographic analysis for the Global Burden of Disease Study 2019. The Lancet **396**, 1160–1203 (2020).
22. NCD Risk Factor Collaboration (NCD-RisC). Trends in adult body-mass index in 200 countries from 1975 to 2014: a pooled analysis of 1698 population-based measurement studies with 19·2 million participants. The Lancet **387**, 1377–1396 (2016).
23. Afshin, A., Micha, R., Khatibzadeh, S. & Mozaffarian, D. Consumption of nuts and legumes and risk of incident ischemic heart disease, stroke, and diabetes: a systematic review and meta-analysis. The American Journal of Clinical Nutrition ajcn.076901 (2014) doi:10.3945/ajcn.113.076901.
24. Aune, D. et al. Nut consumption and risk of cardiovascular disease, total cancer, all-cause and cause-specific mortality: a systematic review and dose-response meta-analysis of prospective studies. BMC medicine **14**, 207 (2016).
25. Aune, D. et al. Fruit and vegetable intake and the risk of cardiovascular disease, total cancer and all-cause mortality–a systematic review and dose-response meta-analysis of prospective studies. International Journal of Epidemiology (2016).
26. Bechthold, A. et al. Food groups and risk of coronary heart disease, stroke and heart failure: A systematic review and dose-response meta-analysis of prospective studies. Critical Reviews in Food Science and Nutrition **59**, 1071–1090 (2019).
27. Schwingshackl, L. et al. Food groups and risk of type 2 diabetes mellitus: a systematic review and meta-analysis of prospective studies. European Journal of Epidemiology **32**, 363–375 (2017).
28. Schwingshackl, L. et al. Food groups and risk of colorectal cancer. International Journal of Cancer **142**, 1748–1758 (2018).
29. Global BMI Mortality Collaboration, E. Di et al. Body-mass index and all-cause mortality: individual-participant-data meta-analysis of 239 prospective studies in four continents. Lancet (London, England) **388**, 776–86 (2016).
30. Singh, G. M. et al. The Age-Specific Quantitative Effects of Metabolic Risk Factors on Cardiovascular Diseases and Diabetes: A Pooled Analysis. PLOS ONE **8**, e65174 (2013).
31. Micha, R. et al. Etiologic effects and optimal intakes of foods and nutrients for risk of cardiovascular diseases and diabetes: Systematic reviews and meta-analyses from the Nutrition and Chronic Diseases Expert Group (NutriCoDE). PLOS ONE **12**, e0175149 (2017).
32. GBD 2017 Diet Collaborators, A. et al. Health effects of dietary risks in 195 countries, 1990-2017: a systematic analysis for the Global Burden of Disease Study 2017. Lancet (London, England) **0**, (2019).
33. Schwingshackl, L. et al. Perspective: NutriGrade: A Scoring System to Assess and Judge the Meta-Evidence of Randomized Controlled Trials and Cohort Studies in Nutrition Research. Advances in Nutrition: An International Review Journal **7**, 994–1004 (2016).
34. World Cancer Research Fund/American Institute for Cancer Research. Diet, Nutrition, Physical Activity and Cancer: A Global Perspective. Continuous Update Project Expert Report. (2018).
35. Aune, D., Norat, T., Romundstad, P. & Vatten, L. J. Dairy products and the risk of type 2 diabetes: a systematic review and dose-response meta-analysis of cohort studies. The American Journal of Clinical Nutrition **98**, 1066–1083 (2013).
36. Aune, D. et al. Dairy products and colorectal cancer risk: a systematic review and meta-analysis of cohort studies. Annals of Oncology: Official Journal of the European Society for Medical Oncology **23**, 37–45 (2012).
37. Mohan, D. et al. Associations of Fish Consumption With Risk of Cardiovascular Disease and Mortality Among Individuals With or Without Vascular Disease From 58 Countries. JAMA Internal Medicine (2021) doi:10.1001/jamainternmed.2021.0036.
38. Poore, J. & Nemecek, T. Reducing food's environmental impacts through producers and consumers. Science **360**, 987–992 (2018).

39. Rennert, K. et al. Comprehensive evidence implies a higher social cost of CO2. Nature **610**, 687–692 (2022).
40. Leal, J., Luengo-Fernández, R., Gray, A., Petersen, S. & Rayner, M. Economic burden of cardiovascular diseases in the enlarged European Union. European Heart Journal **27**, 1610–1619 (2006).
41. Springmann, M., Godfray, H. C. J., Rayner, M. & Scarborough, P. Analysis and valuation of the health and climate change cobenefits of dietary change. Proceedings of the National Academy of Sciences **113**, 4146–4151 (2016).
